# Knowledge Graphs for COVID-19: An Exploratory Review of the Current Landscape

**DOI:** 10.3390/jpm11040300

**Published:** 2021-04-14

**Authors:** Avishek Chatterjee, Cosimo Nardi, Cary Oberije, Philippe Lambin

**Affiliations:** 1 The D-Lab, Department of Precision Medicine, GROW-School for Oncology, Maastricht University, 6229 ER Maastricht, The Netherlands; c.oberije@maastrichtuniversity.nl (C.O.); philippe.lambin@maastrichtuniversity.nl (P.L.); 2 Department of Experimental and Clinical Biomedical Sciences, University of Florence, 50134 Florence, Italy; cosimo.nardi@unifi.it

**Keywords:** COVID-19, knowledge graph, natural language processing, drug repurposing

## Abstract

Background: Searching through the COVID-19 research literature to gain actionable clinical insight is a formidable task, even for experts. The usefulness of this corpus in terms of improving patient care is tied to the ability to see the big picture that emerges when the studies are seen in conjunction rather than in isolation. When the answer to a search query requires linking together multiple pieces of information across documents, simple keyword searches are insufficient. To answer such complex information needs, an innovative artificial intelligence (AI) technology named a knowledge graph (KG) could prove to be effective. Methods: We conducted an exploratory literature review of KG applications in the context of COVID-19. The search term used was “covid-19 knowledge graph”. In addition to PubMed, the first five pages of search results for Google Scholar and Google were considered for inclusion. Google Scholar was used to include non-peer-reviewed or non-indexed articles such as pre-prints and conference proceedings. Google was used to identify companies or consortiums active in this domain that have not published any literature, peer-reviewed or otherwise. Results: Our search yielded 34 results on PubMed and 50 results each on Google and Google Scholar. We found KGs being used for facilitating literature search, drug repurposing, clinical trial mapping, and risk factor analysis. Conclusions: Our synopses of these works make a compelling case for the utility of this nascent field of research.

## 1. Introduction

Due to the truly global nature of the COVID-19 pandemic, there has been an explosion of academic literature on this subject in 2020. Faced with a mountain of data, we often turn to machines for analysis. What we are really after is extracting knowledge from these data, and despite their prodigious computational power, machines are unable to understand, let alone answer, our complex questions. Even for the most basic questions, the search has too often been reduced to keywords, which reduces the sophistication of the query, i.e., loss of semantics. A human easily knows the difference between “Which drugs reduce the severity of COVID-19” and “Which drugs increase the severity of COVID-19”, but for a purely keyword-based search with no semantics, this difference cannot be conveyed to a machine. Now imagine a question such as “Which are the top 3 drugs being trialed for treating COVID-19 in terms of total number of enrolled patients” or “For which clinical outcome is predictive modeling for COVID-19 most successful”? If a machine could answer such questions, it would significantly accelerate scientific progress by providing answers to complex questions that may today require many hours of reading, even by subject matter experts. A knowledge graph (KG) is an artificial intelligence (AI) innovation to bring us closer to this vision [1].

We aimed to conduct a thorough review of how KGs have so far been used in the context of COVID-19. This review should facilitate the widespread use of the existing KGs, and allow researchers to identify the unmet clinical needs, and refocus efforts to produce graphs that actually add to the existing corpus, rather than merely duplicating efforts of other research groups.

## 2. Materials and Methods

Before delving into the methods used for this study, we would like to (1) emphasize that this is not a systematic review, but an exploratory literature review, and (2) explain the reason for making this choice. A defining feature of a systematic review is that it uses a repeatable analytical method to answer a well-defined research question. This translates to using databases such as PubMed, MEDLINE, Web of Science, and clinical trial registries, and having pre-defined inclusion criteria that should ideally be formulated into a study protocol and published before the review starts. Systematic reviews are a great way of synthesizing various information sources in a mature discipline to guide evidence-based medicine. They are often meant to be an exhaustive summary of available evidence, where evidence is defined as peer-reviewed literature indexed in the databases mentioned above. A great example would be a systematic review of the clinical effectiveness of proton therapy. Since a systematic review is often meant to inform clinical practice, the inclusion criteria are much stricter than what is permissible in an exploratory review.

An exploratory review, by contrast, is not meant to follow a repeatable analytical method or be an exhaustive summary. It typically provides a broad overview of work that has been carried out in a certain research domain and uses this to define the scope of future research. In other words, it is meant to define research objectives rather than change or inform clinical practice. Almost every original research paper begins with a short exploratory review of the current state-of-the-art. By its very nature, KGs for COVID-19 is a new field of research. While peer-reviewed literature does exist in this field, there is work being carried out both in academia and in industry that has yet to be published in journals. Thus, using only indexing databases such as PubMed is an inadequate way to capture the current extent of the research.

The aim of this review is to identify the different applications of KGs with respect to COVID-19, even if such research is not mature enough to have been published in peer-reviewed journals that are indexed by PubMed, which is focused on biomedical journals. This includes peer-reviewed articles in non-indexed journals, peer-reviewed conference proceedings, non-peer-reviewed preprints, as well as projects that are described or documented on websites but do not have any published manuscript. For example, the first citation used in this paper cannot be found using PubMed, because the source, i.e., Harvard Data Science Review, published by MIT Press online, is not indexed on PubMed. Unlike PubMed, Google Scholar is not limited to clinical and biomedical journals, and includes conference proceedings, books, and reports, that are not included in Web of Science or PubMed. Google Scholar searches the full text of articles but PubMed and Web of Science search only the citation, abstract, and tagging information. The superiority of Google Scholar over PubMed with respect to the ability to retrieve relevant articles using a quick search has been studied before [2]. The advantages of PubMed over Google Scholar, which mainly stem from PubMed using human curation, are less relevant for this review, because the sources identified by Google Scholar are curated by us before inclusion in the results. Nonetheless, we included PubMed in our search strategy to widen the scope. It would be unacceptable to use Google Scholar for a systematic review because the process must be repeatable, and human judgement used for quality evaluation is subjective and thus not repeatable. However, for an exploratory review, this does not pose a problem, and using Google Scholar allows access to a larger number of sources, sometimes referred to as “grey literature”. An up-to-date comparison of these different search approaches from the perspective of a librarian can be found elsewhere [3].

The search term used for finding original sources for this review was “covid-19 knowledge graph”, and the search was conducted on 7 January 2021 using Google, Google Scholar, and PubMed. The reason for using Google in addition to Google Scholar was to identify companies or consortiums that are working in this field but have not published any literature, peer-reviewed or otherwise. The first five pages of results were considered in both these platforms. Domain expertise was used to reduce this to unique sources, which were then used to obtain the results. This reduction consisted of removing duplicates, verification of the relevance, and qualitative assessment of the rigor of publicly accessible work, whether in the form of articles or websites. The Results section constitutes a broad overview of the field; it separates the KGs for COVID-19 into clusters based on their intended use, and then briefly summarizes the information pertaining to each original source. No KG-related paper that was discovered in the search was rejected from inclusion. There was no a priori decision on how many papers to include under each type of KG application. The Results section does not include any subjective opinions from us; such opinions are presented in the Discussion section.

## 3. Results

Our search yielded 34 results on PubMed and 50 results each on Google and Google Scholar, as the default number of results per page on both these platforms is 10. Table 1 summarizes the papers we found and the main application they reported for the KGs they created. In our review, the papers are clustered into *application groups* depending on their KG application. These application groups and their associated papers are described in the rest of this section. In addition, our search also pointed us to the EU Datathon 2020 which organized two meetups of The Knowledge Graph Conference in April. The associated recordings and slides can be found in the following link (accessed on 7 January 2021). We also found the CovidGraph project (https://covidgraph.org) (accessed on 7 January 2021), an interdisciplinary collaboration between academia and industry. In addition to the literature data, they connected information from genes and proteins and their function, using open-source knowledge bases such as the Gene Ontology and the NCBI Gene Database. An important advantage of this project is that it uses Neo4j [4] for modeling, storing, and exposing the KG, which considerably simplifies adoption by a large body of data scientists and app developers, as it is both powerful and intuitive. However, since there is no paper associated with this project yet, we cannot provide further detail in this review.

### 3.1. Knowledge Graphs for Literature Search

We found four articles that used KGs to facilitate the literature search. In the first paper by Steenwinckel et al. [5], the Kaggle dataset of 63,000 + papers (also known as CORD-19 [6], released to allow recent advances in natural language processing (NLP) and other AI techniques to generate new insights to fight the pandemic) was used to create a KG. The authors started with a summary of initiatives by other research groups who are using the same dataset, identifying the CovidGraph project as the largest such initiative. The authors then discussed the steps needed to construct their KG. In the CORD-19 dataset, information about each paper is provided as a CSV file. For over 51,000 of these papers, JSON files exist, containing information about the authors, the content, and the other cited studies. To semantically enrich the data, the authors mapped them to the resource description framework (RDF) using the RDF mapping language (RML), which was convenient because the initial data were already structured (CSV and JSON). Before the conversion from JSON to RDF, the JSON files were extended to include additional information from external resources, including DBpedia [7], BioPortal [8], CrossRef [9], and ORCID [10]. 

To make the transformation from JSON to RDF, a mapping document was created that contained rules on how each element in the JSON can be mapped on a corresponding semantic output value. The mapping document was created with YARRRML, a human-readable text-based representation that can be used to represent RML rules [11]. This YARRRML document was then converted to an RML document by using the YARRRML Parser. The reason for using the intermediate YARRRML step instead of writing RML rules directly was because YARRRML, being human-readable, allows other researchers to extend the mapping documents with less effort and without needing expertise in semantic web formats. The RMLMapper [12], using both the extended JSON files and the RML document as input, produces a set of *N*-Triples for each paper. The authors concatenated all such *N*-Triple files to form a single KG.

The authors then discussed the applications of such a KG, which we summarize in the rest of this paragraph. They state that current network analysis tools cannot handle different labeled edges that make up such a multi-relational KG. Thus, the KG needs to be converted to a regular directed graph by retaining only citation information to perform network analysis. Nodes of their converted graph represent the papers; graph edges represent citations from one paper to another. The interconnectedness of publications via citations can be revealed using clustering analysis. Node centrality analysis can identify publications that are influential with respect to COVID-19, rather than influential in general, for which looking at number of citations would suffice; the centrality of a node can be quantified via different metrics. Machine learning cannot be directly applied to KGs. As a workaround, knowledge graph embeddings can be used, where KG components, including entities and relations, are embedded into continuous vector spaces. RDF2vec [13] is the most common technique to build such embeddings. Once converted into these vectors, one can search for nearest neighbors to find similar or related papers in a much more powerful way than a keyword search. These vectors can also be used for clustering papers, which is more powerful than the network clustering analysis previously described, which only uses citation links.

**Figure 1:**
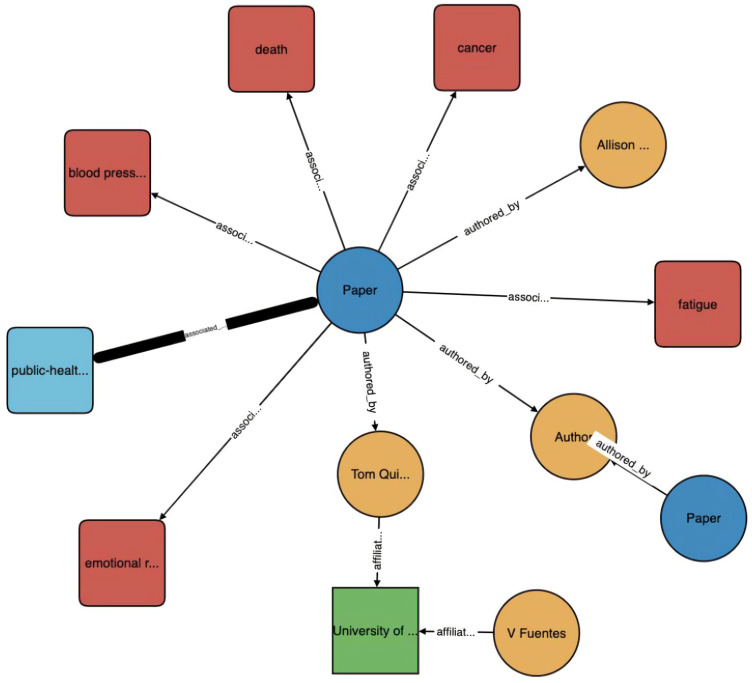
Visualization of a knowledge graph (KG). Paper entities (blue) connect to concepts (red), topics (light blue), and authors (gold) through directed relations. Authors connect to institutions (green). Reproduced with permission from Wise et al. [14].

The second paper, by Wise et al. [14], also uses the CORD-19 dataset, is a demonstration of Amazon Web Services AI, and is conceptually more advanced than the first paper. Unlike the first paper, the second paper does not support the FAIR (findable, accessible, interoperable, and reusable) principle, and does not make any of its code public. However, their KG is used to power a search engine (https://www.cord19.aws/) (accessed on 7 January 2021), which is available for public use. The authors provide a succinct definition of a KG: “Knowledge graphs (KGs) are structural representations of relations between real-world entities where relations are defined as triplets containing a head entity, a tail entity, and the relation type connecting them.” Their KG contains five types of entities: paper (with attributes of title, publication date, journal, and digital object identifier (DOI) link), author (with attributes of first, middle, and last names), institution (with attributes of name, country, and city), concept, and topic. Figure 1 illustrates the directed property graph structure for a small subgraph of their KG.

*Concept entity*: They used their proprietary NLP system named Comprehend Medical Detect Entities V2 for medical language entity recognition and relationship extraction. Given the example text “Abdominal ultrasound noted acute appendicitis, recommend appendectomy followed by several series of broad spectrum antibiotics”, the system extracts Abdominal (Anatomy), ultrasound (Test Treatment Procedure), acute appendicitis (Medical Condition), appendectomy (Test Treatment Procedure), and antibiotics (Medication) as recognized entities along with entity types and model confidence scores. Entity names, e.g., acute appendicitis, form concept entities while entity type and model confidence score are the entities’ attributes. *Topic entity*: They defined 10 topics using expert knowledge: Vaccines/Immunology, Genomics, Public Health Policies, Epidemiology, Clinical Treatment, Virology, Influenza, Healthcare Industry, Lab Trials (human) and Pulmonary Infections. Since manually labeling a topic model is inefficient, they manually labeled only a subset of the papers and used this to train a multi-label classifier, an extension of Latent Dirichlet Allocation termed Z-LDA, using the title, abstract and body text from each paper. The resulting classifier achieved an average F1-score of 0.92 with on average of 2.37 labels per document. To validate their topic model, they checked that generated topics of papers from Journal of Virology, e.g., virology, genomics, and lab-trials-human, were highly related to virology and the generated topics of papers from Journal of Vaccine, e.g., vaccines-immunology, were highly related to vaccinology.

To curate their KG, they applied data normalization techniques which eliminated duplicate entities and noisy linkages. Denoising included thresholding on the confidence scores, pruning concepts that occur in less than 0.0001% of papers, and flagging concepts that appear in greater than 50% of papers for manual assessment. The KG was then used for two main tasks: information retrieval and article recommendations. For information retrieval, an example query “What papers discussing COVID-19 risk factors are most often cited by researchers within the CORD-19 dataset?” results in two steps: first, the articles which contain the risk factors as entities are retrieved, and then these articles are ranked based on citation counts within the dataset. The authors combined article semantic information with KG topological information to quantify similarity between articles and construct a similarity-based recommendation system: given a paper, the engine retrieves a list of top-k most similar papers using cosine distance. To capture semantic information, they used SciBERT [15] that has shown strong transfer learning performance on a wide variety of NLP tasks. To capture KG topological information, they generated vector embeddings for each paper by using the algorithm TransE [16] and Deep Graph Library Knowledge Embedding library (DGL-KE [17]). Besides finding similar papers to a given paper, the recommendation engine can also be used to identify the most popular papers, where popularity captures the number of occurrences of an individual paper in the top-5 most similar items list for all papers in the dataset.

The third paper, by Cernile et al. [18], also uses the CORD-19 dataset, and makes the dataset and visualizations publicly accessible via a webtool. They used proprietary NLP and AI engines which leverage a fast heuristic search algorithm and a knowledge-driven approach for concept identification, context determination, inferencing and extraction of corresponding values and units. The study used a collection of 10 general knowledge bases and nine domain-specific knowledge bases that were built using UMLS (Unified Medical Language System) terms and updated with recently added terms specific to COVID-19. Generic terms with little significance were determined, for example, “air”, “water” and “virus”, and removed from the set of extracted concepts. For each term found in a paper, a link was created to every other term in the same paper. The summation of these links over all papers determined the weight of a connection between any two terms: the number of papers linking the terms. Additional filtering was performed to refine the scope of the network and removal of noise to aid readability and navigation; for example, links with low weights were removed, as were links with terms that were disconnected from the rest of the network. Network nodes were colored based on the knowledge source, with the size of the nodes proportional to the frequency of each term and the connection weight, i.e., edge thickness, based on the number of associated papers. Four network graphs were generated using these extracted data: cardiological diseases, lung diseases, title network and treatment network (https://nlp.inspirata.com/networkvisualisations/treatmentnetwork/) (accessed on 7 January 2021).

The fourth paper, by Michel et al. [19], has grander ambitions than just a literature search. The COVID-on-the-Web Dataset created by this team can be put to other uses in the future, such as creating argumentative graphs which can be used by clinicians to analyze clinical trials for evidence-based decision-making. We categorized Michel et al. under a literature search as this is what is explicitly demonstrated in their current work. The authors are strong proponents of open and reproducible science goals, and the FAIR principles. Like the previous papers mentioned in this subsection, they also used the CORD-19 dataset, and enriched it using DBpedia, BioPortal, and Wikidata to create the *CORD-19 Named Entities Knowledge Graph*. In addition, all CORD-19 abstracts were analyzed by argumentative clinical trial analysis (ACTA [20]) and translated into RDF to create the *CORD-19 Argumentative Knowledge Graph*. ACTA is designed to analyze clinical trials to extract argumentative components and PICO (patients/population (P), intervention (I), control/comparison (C) and outcome (O)) elements. Finally, they provided several visualization and exploration tools based on the Corese Semantic Web platform (https://project.inria.fr/corese/) (accessed on 7 January 2021) and MGExplorer visualization library (https://github.com/frmichel/morph-xr2rml/) (accessed on 7 January 2021).

ACTA retrieves the main claim(s) stated in the trial, the evidence linked to this claim, and the PICO elements. For a clinical trial, a claim is a concluding statement made by the author about the study outcome. It generally describes the relation of a novel treatment with respect to existing treatments, referred to as intervention arm and control arm, respectively. An observation or measurement is evidence, consisting of side effects and the outcome. Two relation types can hold between argumentative components: attack and support, depending on whether a statement or observation is contradicting or justifying the proposition of the target component. The ACTA pipeline comprises four steps: (i) detecting argumentative components, i.e., claims and evidence, (ii) predicting the relations between components, (iii) extracting PICO elements, and (iv) producing the RDF representation of the arguments and PICO elements.

To demonstrate the immediate clinical utility of Michel et al.’s ACTA framework (http://ns.inria.fr/acta/) (accessed on 7 January 2021), we applied it on 20 published papers that are all related to clinical trials for COVID-19 interventions. The papers were chosen by searching clinicaltrials.gov for all completed COVID-19 trials with available results, and then extending the search using The New England Journal of Medicine. The authors included in ACTA the possibility to search for a (set of) abstract(s) directly on the PubMed catalogue through PubMed’s application programming interface (API). When the search results are shown, the user can select one or more abstracts to perform the argumentative analysis. The result is displayed to the user as an argumentative graph where the nodes are the premises and claims automatically detected in the abstract, together with their links. When the user hovers over a node, the textual content of the argumentative component is shown. The full text of the abstract is shown on the right side of the graph, where the user can highlight in different colors either the argumentative components or the PICO elements. The PICO identification results have been included in the Supplementary Material. 

### 3.2. Knowledge Graphs for Drug Repurposing

We found five articles related to using KGs for drug repurposing, which is a technique of using existing drugs to treat emerging and challenging diseases, thereby reducing development timelines and overall costs. The first article, by Stebbing et al. [21], was published as a comment in Lancet Infectious Diseases near the beginning of the pandemic (1 April 2020). The authors had earlier described how BenevolentAI’s proprietary KG, queried by a suite of algorithms, enabled the identification of baricitinib, a numb-associated kinase (NAK) inhibitor, to suppress clathrin-mediated endocytosis and thereby inhibit viral infection of cells. In this work, they re-examined the affinity and selectivity of all the approved drugs in their KG to identify those with both antiviral and anti-inflammatory properties, since the host inflammatory response becomes a major cause of lung damage and subsequent mortality for severe cases of COVID-19. This yielded three candidates: baricitinib, fedratinib, and ruxolitinib. Other AI-algorithm-predicted NAK inhibitors included a combination of the oncology drugs sunitinib and erlotinib, shown to reduce the infectivity of a wide range of viruses. However, sunitinib and erlotinib would be difficult for patients to tolerate at the doses required to inhibit NAK. Baricitinib emerged as the best choice, especially given its once-daily oral dosing and acceptable side-effect profile. In addition, the potential for combination therapy with baricitinib was high, including combining baricitinib with the direct-acting antivirals (lopinavir or ritonavir and remdesivir) currently being used in the COVID-19 outbreak to reduce viral infectivity, viral replication, and the aberrant host inflammatory response. A trial of baricitinib plus remdesivir has already been conducted and was superior to remdesivir alone in reducing recovery time and accelerating improvement in clinical status [22].

The second article, by Wang et al. [23], used KGs for drug repurposing report generation. For a given drug, such a report consists of 11 typical questions they identified: (1) current indication: what is the drug class? What is it currently approved to treat? (2) Molecular structure; (3) mechanism of action, e.g., inhibits viral entry, replication; (4) Was the drug identified by manual or computation screen? (5) Who is studying the drug? (Source/lab name); (6) in vitro data, e.g., cell line used, assays run, viral strain used, cytopathic effects, toxicity, LD50, dosage response curve; (7) animal data, e.g., what animal model, LD50, dosage response curve; (8) ongoing clinical trial data, e.g., what phase, facility, target population, dosing, intervention; (9) funding source; (10) has the drug shown evidence of systemic toxicity? (11) List of relevant sources to pull data from. The summary of their framework can be seen in Supplementary Figure S1.

**Figure 2:**
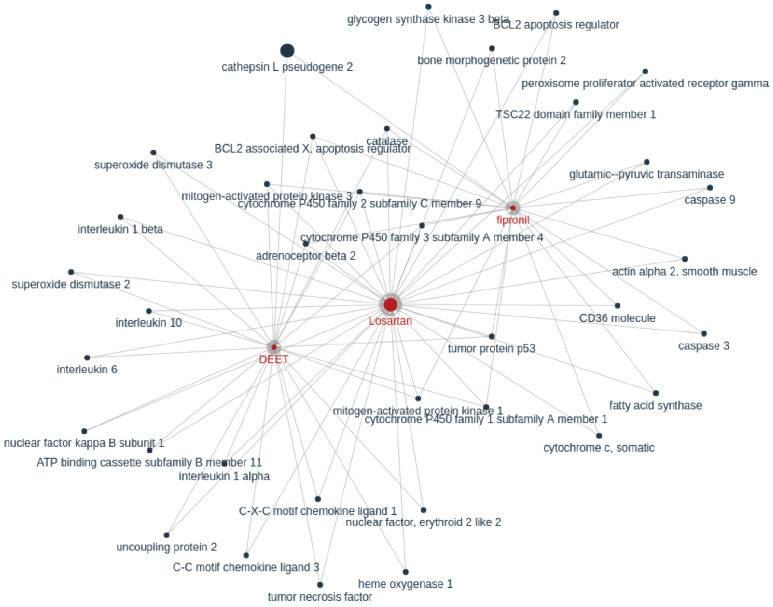
Constructed KG connecting losartan (candidate drug in COVID-19) and cathepsin L pseudogene 2 (gene related to coronavirus). Reproduced with permission from Wang et al. [23].

**Figure 3:**
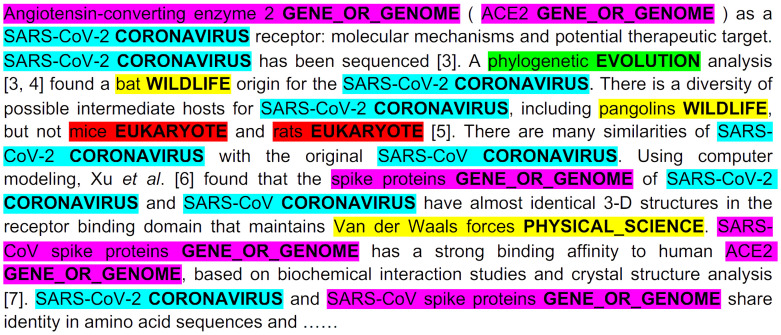
Example of fine-grained entity extraction. Reproduced with permission from Wang et al. [23].

They built a multimedia KG by combining (1) coarse-grained text knowledge extraction, (2) fine-grained text entity extraction, (3) image processing and cross-media entity grounding, and (4) KG semantic visualization. A KG constructed after just step (1) can be seen in Figure 2. A demonstration of steps (2) and (3) can be seen in Figure 3 and Supplementary Figure S2 respectively. Step (4) enhances the exploration and discovery of the information in the KG by allowing user interactivity that surpasses directed keyword searches or simple unigram word cloud or heatmap displays. Several clinicians and medical school students in their team reviewed the drug repurposing reports for three drugs that were used as a case study for the paper (benazepril, losartan, and amodiaquine), and also the KGs connecting 41 drugs and COVID-19-related chemicals/genes. Preliminary results show that most of their output was informative and valid.

The third article, by Domingo-Fernandez et al. [24], created a KG that is a cause-and-effect knowledge model of COVID-19 pathophysiology, which could then be applied for drug repurposing. The authors point out that although KGs were originally developed to describe interactions between entities, novel machine learning techniques can generate latent, low-dimensional representations of the KG which can then be utilized for downstream tasks such as clustering or classification. For the creation of the KG, scientific literature related to COVID-19 was retrieved from open access and freely available journals: PubMed, Europe PMC, and additional COVID-19 specific corpuses such as LitCovid. This corpus was then filtered based on available information about potential drug targets for COVID- 19, biological pathways in which the virus interferes to replicate in its human host, and information on the various viral proteins along with their functions. Finally, the articles were prioritized based on the level of information that could be captured in the modeling language used to build the KG. Evidence text from the prioritized corpus was manually encoded in biological expression language (BEL) as a triple including metadata about the nodes and their relationships as well as corresponding provenance and contextual information. BEL involves encoding mechanistic information such as protein–protein interactions, observed correlations between phenotypes and molecules, or effect of drugs on a given target. Therefore, only BEL encodable articles were selected. The authors explained in the Supplementary Material why they favored this manual curation over a text-mining approach, arguing that the manual approach provides better quality in terms of contextualization, i.e., finding the proper relation between two entities due to the complexity of scientific writing, and the understandability of the KG. They mentioned the possibility of using a semi-automatic pipeline to combine the advantages of manual curation and text-mining.

Their KG summarizes mechanistic information on COVID-19 published in 160 original research articles. As described in their publication, the COVID-19 KG incorporates 4016 nodes, covering 10 entity types (e.g., proteins, genes, chemicals, and biological processes) and 10,232 relationships (e.g., increases, decreases and association). They mentioned that given the selected corpora, these cause-and-effect relations primarily denote host–pathogen interactions as well as comorbidities and symptoms associated with COVID-19. Furthermore, the KG contains molecular interactions related to host invasion (e.g., spike glycoprotein and its interaction with the host via receptor ACE2) and the effects of the downstream inflammatory, cell survival and apoptosis signaling pathways. The authors have identified over 300 candidate drugs currently being investigated in the context of COVID-19, including proposed repurposing candidates and drugs under clinical trial.

The fourth paper, by Hsieh et al. [25], aimed to discover repurposable drugs by integrating multiple SARS-CoV-2 and drug interactions, deep graph neural networks (GNN), and in vitro/population-based validations. They collected all the available drugs (*n* = 3635) involved in COVID-19 patient treatment through the Comparative Toxicogenomics Database. The candidate drugs can be divided into two broad categories: those that can directly target the virus replication cycle, and those based on immunotherapy approaches either aimed to boost innate antiviral immune responses or to alleviate damage induced by dysregulated inflammatory responses. They built a SARS-CoV-2 KG based on the interactions among virus baits, host genes, drugs, and phenotypes. The graph had four types of nodes and five types of edges based on the interactions. The four types of nodes include 27 virus baits, 5677 unique host genes, 3635 drugs, and 1285 phenotypes. The five types of edges include 330 virus–host protein–protein interactions, 13,423 pairwise genes on the same pathway, 16,972 drug-target pairs, 1401 gene–phenotype pairs, and 935 drug–phenotype pairs.

A GNN approach was used to derive the candidate drug’s representation based on the biological interactions. To justify their approach, the authors explained that in traditional network analysis, network proximity is defined with direct interactions, thus a node’s local role (e.g., neighbors, edge directions) and global position (e.g., overall topology or structure) are less considered. GNNs derive a vectorized representation (i.e., embedding) of nodes, edges, or whole graphs. The graph node embeddings used by a GNN can preserve the node’s local role and global position in the graph via iterative and nonlinear message passing and aggregation. A GNN learns the structural properties of the neighborhood and the graph’s overall topological structure. The graph embedding method used was the variational graph autoencoder with multi-relational edges. The authors prioritized the candidate drugs using clinical trial history, and then validated them with their genetic profiles, in vitro experimental efficacy, and electronic health records. The top 22 drugs included azithromycin, atorvastatin, aspirin, acetaminophen, and albuterol. They further pinpointed drug combinations that may synergistically target COVID-19, including hydroxychloroquine plus melatonin.

The fifth article, by Zhou et al. [26], is a review article for Lancet Digital Health. In the review, the authors introduced guidelines on how to use various forms of AI for accelerating drug repurposing, with COVID-19 as an example. With regard to KGs in particular, they mention that KGs can be reduced to low-dimensional feature vectors, and using the feature vectors of drugs and diseases, one can then measure their similarities and thus identify effective drugs for a given disease. One challenge they identify for the graph embedding method is scalability. The number of entities in a medical KG could be as many as several million. They mention that several systems have been specifically designed for learning representations from large-scale graphs (e.g., GraphVite [27]). The authors identified two works which evaded our search strategy: Gysi et al. ([28], which did not use the term *knowledge graph* in the paper) and Zeng et al. [29]. Zeng et al.’s KG included 15 million edges across 39 types of relationships connecting drugs, diseases, proteins, genes, pathways, and expressions of genes and proteins from a large scientific corpus of 24 million PubMed publications. Using Amazon Web Services’ computing resources and graph representation learning techniques (DGL-KE, mentioned earlier in this paper in the context of literature search), they identified 41 repurposable drug candidates including dexamethasone, thalidomide, and melatonin whose therapeutic associations with COVID-19 were validated by transcriptomic and proteomics data in SARS-CoV-2-infected human cells and data from ongoing clinical trials.

### 3.3. Knowledge Graphs for Clinical Trials

**Figure 4:**
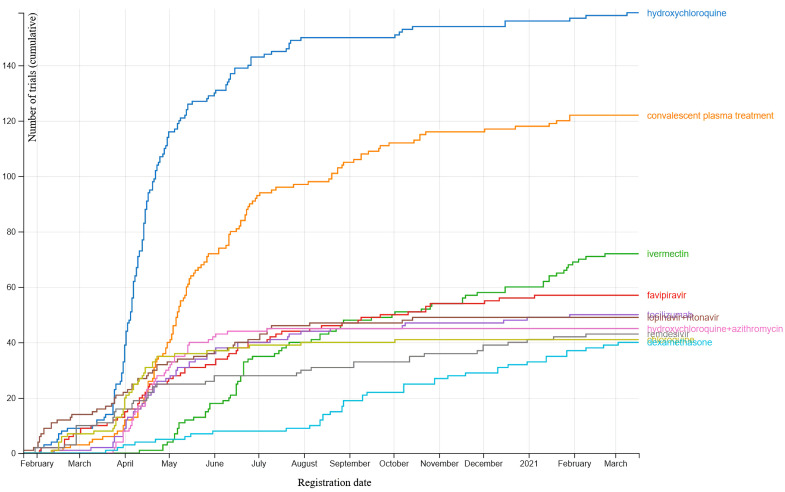
Trend of number of trials registered by treatment name. Taken from https://covid-nma.com/dataviz (accessed on 25 March 2021).

**Figure 5:**
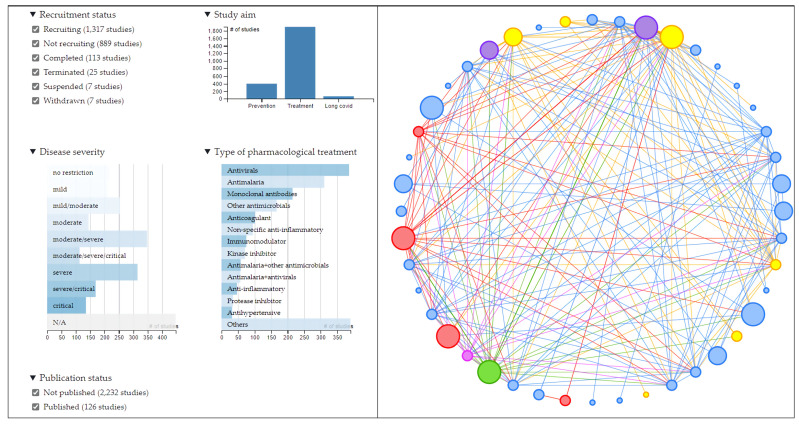
The diagram on the right describes the network of randomized control trials (RCTs) evaluating pharmacologic treatments for COVID-19 which fulfill criteria that the user selects. The nodes in the diagrams represent the different treatments evaluated in these RCTs and the lines represent the direct comparisons made in the studies. When two nodes are connected with a line, it means there is at least one study that compares the corresponding treatments, whereas when they are not connected, it means there is no study comparing them. The size of the nodes is proportional to the number of participants allocated to each intervention and the thickness of the lines is proportional to the number of studies that compare each pair of treatments. Taken from https://covid-nma.com/dataviz (accessed on 23 February 2021).

**Figure 6:**
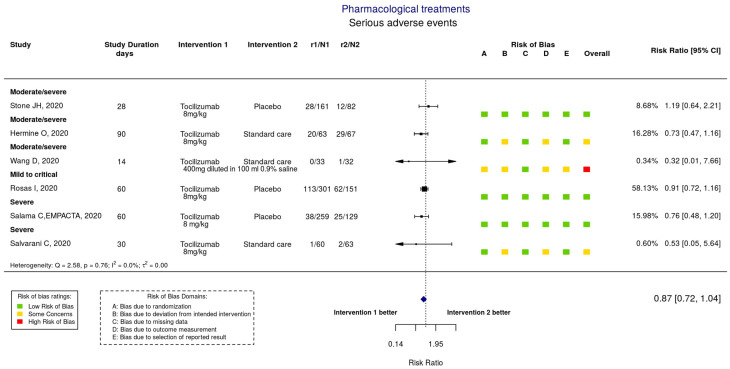
A forest plot comparing two interventions chosen by the user, in this case tocilizumab and placebo/standard of care. Taken from https://covid-nma.com/dataviz (accessed on 23 February 2021).

The pre-eminent effort to synthesize the results of clinical trials related to the prevention and treatment of COVID-19 is the COVID-NMA initiative (https://covid-nma.com/) (accessed on 25 March 2021). This project aims to provide a complete, high-quality, and up-to-date synthesis of evidence as soon as results are available as well as a living mapping of registered randomized controlled trials. The vast majority of work involved in curating the database is carried out by human volunteers. This synthesis will allow evidence-based decision-making and planning of future research. We would like to mention that this initiative cannot be classified as a KG in the AI sense, because the concept of triples, which is central to an AI KG, is not used. However, we still include information about this initiative in this review because the results of this approach are exactly in line with the goals of a KG. The living mapping of trials registered on the WHO platform is updated weekly and contained 2358 randomized control trials (RCTs) at the end of 2020. The living synthesis of published trials, including both articles and preprints, is updated daily, and contained 157 RCTs with results at the end of 2020. The highly interactive data visualizations that have been developed as a result of this initiative constitute some of the most useful summaries of COVID-19 research. Some examples are shown in Figure 4, Figure 5 and Figure 6, but to fully appreciate the flexibility provided by the visualization tools, we encourage the reader to visit the website. Potentially this high-quality human curation can be replaced in the future by AI to ensure sustainability. 

### 3.4. Multi-Purpose Knowledge Graphs

We found three papers that use KGs for multiple tasks, including literature search and drug repurposing. The first, by Chen et al. [30], carried out a case study on the application possibilities of KGs. The introduction of their paper provides an excellent history of the emergence of KGs in the field of AI, which we summarize in the rest of this paragraph. They point out that in the past, KGs have been curated manually, but the move towards natural language understanding through semantic technologies has accelerated in the past decade, promoting named entity recognition (NER) to a central NLP task. NER has been crucial for building and constructing KGs as the primary method of analyzing free text to extract entities and possibly relations. Additionally, tasks such as link prediction, relation extraction, and graph completion on KGs are aided by NER. In the early 2000s, biomedical NER relied on feature engineering and graphical models such as hidden Markov models (HMMs) and conditional random fields (CRFs), which had poor accuracy compared to the current state-of-the art which uses deep learning. Bidirectional Encoder Representations from Transformers (BERT [31]) is the foundational work from Google that has made deep-learning-based NER possible. BioBERT [32] is a biomedical language representation model based on BERT used by the authors to mine the CORD-19 dataset, as well as the PubMed database and PubMed KG. 

**Figure 7:**
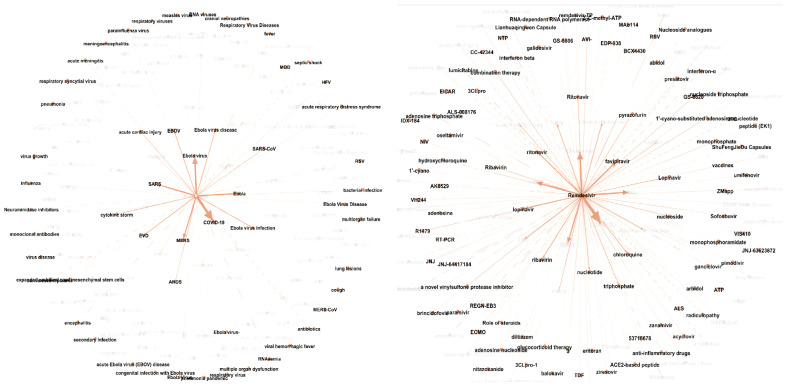
Remdesivir-related KGs: associated diseases (left) and associated drugs (right) based on co-occurrence frequency. Reproduced with permission from Chen et al. [30].

To illustrate the utility of KGs, the authors performed several experiments, the most basic of which was compiling a list of most-published authors in the CORD-19 dataset. In an experiment using BioBERT, they found that BioBERT can easily recognize the common bio-entities with a high occurrence rate in the corpus but fails to recognize rare biomedical terms. They used two metrics to find the strength of KG associations, i.e., weights, between source and target nodes: co-occurrence frequency and cosine similarity. Figure 7 shows KGs related to remdesivir based on co-occurrence frequency. They state that while this is a promising approach, a major limitation of co-occurrence frequency is that it cannot reflect the relationship between the source node and the target node well. For example, if “A has nothing to do with B” is mentioned often in documents, its co-occurrence frequency will be high. Cosine similarity has the benefit of being a normalized metric unlike co-occurrence frequency, but it still has the same limitation.

The second paper, by Reese et al. [33], is a framework for producing KGs that can be customized for downstream applications including machine learning tasks, hypothesis-based querying, and browsable user interface. For example, a drug repurposing application would make use of protein data linked with approved drugs, while a biomarker application could utilize data on gene expression linked with pathways. The authors explain that researchers are confronted with a number of technical challenges when trying to use existing data to discover actionable knowledge about COVID-19, which we summarize in the rest of this paragraph. The data needed to address a given question are typically siloed in different databases and employ different identifiers, data formats, and licenses. For example, to examine the function of proteins targeted by FDA-approved antiviral drugs, one must download and integrate drug, drug target, and FDA approval status data (from Drug Central, for example, in a bespoke TSV format) and functional annotations (from, for example, Gene Ontology in GPAD format). Furthermore, many datasets are updated periodically, which requires researchers to re-download and re-harmonize data. KGs are a way of representing and integrating heterogeneous data and their interrelationships using a hierarchical system such as an ontology. This kind of representation is amenable to complex queries, e.g., “which drugs target a host protein that interacts with a viral protein?”, and also to graph-based machine learning techniques.

Their workflow is divided into three steps: data download (fetch the input data), transform (convert the input data to KGX interchange format), and merge (combine all transformed sources). The ingested data are focused on sources relevant to drug repurposing for downstream querying and machine learning applications, prioritizing drug databases, protein interaction databases, protein function annotations, COVID-19 literature, and related ontologies. From the final merged graph, training and test data sets suitable for machine learning applications are created. Embiggen, their implementation of node2vec and related algorithms, is applied to this KG to generate embeddings, vectors in a low dimensional space which capture the relationships in the KG. Embiggen is trained iteratively to identify optimal node2vec hyperparameters (walk length, number of walks, *p* etc.) and to then train classifiers, e.g., logistic regression, random forest, support vector machines, that can be used for link prediction. The trained classifiers can then be applied to produce actionable knowledge: drug to disease links, drug to gene links, and drug to protein links. Besides machine learning, the authors have also used the KG for hypothesis-based querying. For example, they have queried the KG to identify host proteins that are known to interact with viral proteins, and these are further filtered according to whether these host proteins are targets of approved drugs. In the framework created by the authors, each data source is transformed and output as a separate graph, which is later combined with graphs for other data sources according to the needs of the user. They explain that although the subgraphs from the various data sources, e.g., Drug Central, are produced locally by their framework, they could easily incorporate graphs generated by other researchers. The exchange of data via a ‘KG-Hub’ would eliminate the duplication of effort that occurs when researchers separately transform and prepare data and might also facilitate the formation of a data sharing portal.

The third article, by Ostaszewski et al. [34], describes a large-scale community effort to build an open-access, interoperable, and computable repository of SARS-CoV-2 virus–host interaction mechanisms. They discuss the tools, platforms, and guidelines necessary for the distributed development of this Disease Map (a constantly evolving collection of machine-and-human-readable computational diagrams and models of molecular mechanisms implicated in the disease) by a community of biocurators, domain experts, bioinformaticians, and computational biologists. Biocurators develop a collection of systems biology diagrams focused on the molecular mechanisms of SARS-CoV-2. Domain experts refine the contents of the diagrams, supported by interactive visualization and annotations. Using interaction and pathway databases (which contain structured and annotated information on protein interactions or causal relationships) and text mining, they enrich and validate the curated mechanisms. The authors use text mining and pathway figure mining, i.e., decoding pathway figures into their computable representations, to create KGs, which they define as “semantic networks incorporating ontology concepts, unique biomolecule references, and their interactions extracted from abstracts or full-text documents”. Biocurators can then use this content: by visual exploration, by programmatically querying the KGs, and by direct incorporation of the content after converting to the appropriate file format. Bioinformaticians and computational biologists develop computational workflows to generate hypotheses and predictions about the mechanisms encoded in the diagrams. The Disease Map provides a platform for a precise formulation of mechanistic models, accurate data interpretation, monitoring of therapy, and potential for drug repositioning.

### 3.5. Knowledge Graphs for Risk Factor Discovery

We found only one paper of this type, and thus feel this is an under-explored application of KGs. Bettencourt-Silva et al. [35] present a pipeline to discover COVID-19 health outcomes and related social factors based on trending social determinants of health (SDoH) at population-level using Google Trends. SDoH are the factors which lie outside of the traditional health system, such as employment or access to nutritious foods, that influence health outcomes. The authors point out that electronic health record systems have not traditionally been designed to capture SDoH-related data and healthcare terminologies such as ICD-10 (10th revision of the International Statistical Classification of Diseases and Related Health Problems, a medical classification list by the World Health Organization) or SNOMED-CT (Clinical Terms defined by SNOMED International, an international non-profit standards development organization) may not extensively cover social concepts. A WHO-defined set of SDoH keywords was monitored using Google Trends. Specific SDoH keywords were then identified by performing a statistical analysis of population data, e.g., keywords trending higher in a particular time period, i.e., February to April 2020, compared to historical data. Such keywords became seeded terms to be found as nodes in a KG of related concepts. Finding the nodes connected to the seeded terms by traversing the KG yielded additional nodes with insights of potentially relevant concepts to be investigated further. From the list of ten Google Topics, *Unemployment* and *Food Insecurity* were the two that peaked the most during the start of the pandemic and also saw their highest 5-year peaks in the same period. These two concepts were selected for the case study presented in this paper to illustrate the developed pipeline.

Their KG was built by first mining co-occurring concepts, i.e., a pair of concepts, at least one of which is *Unemployment* or *Food Insecurity*, from the literature. Starting from the PubMed database, the authors used MetaMap to tokenize and identify UMLS concepts in the sentences of the abstracts. They restricted the medical concepts to only those of the following UMLS semantic types: *Disease or Syndrome, Individual Behavior, Mental or Behavioral Dysfunction*. These concepts seemed to be the most relevant to their aim of identifying potential socio-medical issues in the context of COVID-19. They filtered out of the results the sentences containing three concepts or more, which they believed would prove too difficult to use to extract accurate pairwise relations. To extract relations between a concept pair, they used a supervised sentence classification model, a fine-tuned BERT. To train the model, they sampled 550 of the context sentences and manually annotated them with five labels: positive if the concepts were found to be in positive correlation, negative for a negative correlation, complex for a more complex relation not easily classified as the first two (e.g., a relation conditioned on a specific characteristic of the population), nocor if the authors did not find a correlation, and N/A for sentences not expressing any statement on the relation. A graph database was subsequently used to store, query, and visualize the mined concepts.

**Figure 8:**
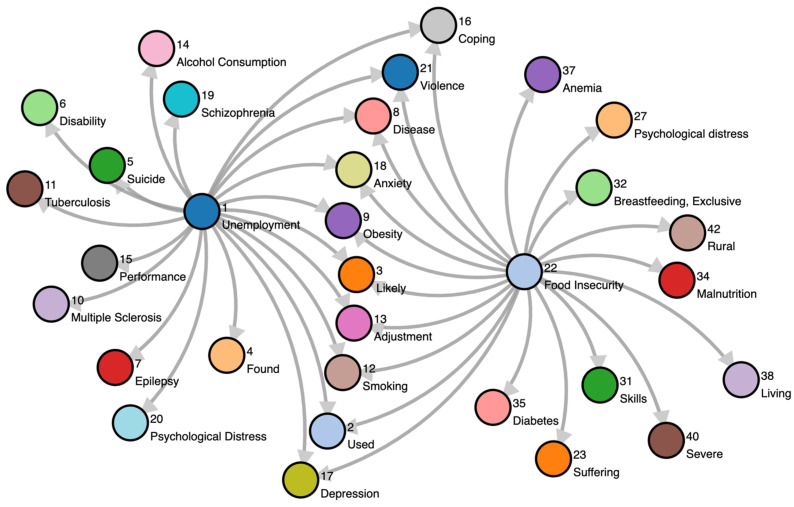
Two social determinants of health (SDoH) dimension concepts (*Unemployment* and *Food Insecurity*) and their most relevant neighbors based on relative frequency. Reproduced with permission from Bettencourt-Silva et al. [35].

The results of their work can be seen in Figure 8, which shows the two SDoH dimension concepts and their most relevant neighbors based on relative frequency. The authors state that the most interesting nodes are the ones connected to both SDoH dimensions (e.g., *Obesity* or *Depression*), and that such concepts should be closely monitored and analyzed in the time period following the start of the pandemic. For example, a simple analysis of Google Trends (Worldwide) from May to June 2020 revealed peaks for *Obesity* (Google Trend class: medical condition) and *Coping* (topic) in May 2020 and for *Anxiety* (emotional disorder) in June. These examples show the largest interest recorded in the past 5 years. They explain that further work is needed to analyze these data, inspect other geographical levels (e.g., country), and understand the causes for the sudden rise in these concepts. Their proposed pipeline should have wider applicability in (a) identifying social or clinical characteristics of interest, (b) outbreak surveillance, or (c) mining relations between social and health concepts that can help inform and support citizen-centered services.

## 4. Discussion

To our knowledge, this is the first exploratory review focusing on KGs that are used to accelerate COVID-19 research. We found KGs being put to different uses, with typically multiple publications per use. In addition, we provided detailed summaries of the methodologies that were used in these publications, making this article interesting not only for researchers and clinicians focusing on COVID-19, but also readers interested in implementing KGs in other domains, including other diseases which have a high volume of research output being generated.

A seeming limitation of this study is that the papers that correspond to the same application group are not compared quantitatively. However, this arises due to the nature of the papers themselves. When trying to evaluate the pros and cons of different approaches that are trying to address the same need, e.g., a literature search using KGs, it is important to have agreed upon benchmarks in terms of datasets used and performance metrics. Such benchmarks do not exist yet, in the absence of which we are forced to compare methods based mainly on methodology. While methodological differences between two approaches can be obvious, the impact that such differences will have on performance cannot be gauged without actually using the methods, which is beyond the scope of this work. Furthermore, the choice of the “best” approach may depend on the end user; for example, when choosing the right search engine to conduct a literature search, if the end user is a developer, they could prefer the approach that offers open-source code that the developer can use as a starting point. If the end user is a clinician, they might prefer the approach that is more user-friendly and offers more relevant results. Keeping these caveats in mind, we now present a comparative assessment of the papers that belong to the same application group, i.e., literature search, drug repurposing, and multi-purpose. Clinical trial mapping and risk factor analysis are excluded from this discussion, as we only found one effort each for these uses.

### 4.1. Knowledge Graphs for Literature Search

Four papers were reported under this application group: Steenwinckel et al. [5], Wise et al. [14], Cernile et al. [18], and Michel et al. [19]. All of these papers used the CORD-19 dataset; [14,18] use proprietary code, whereas [5,19] have made their code open-source and followed FAIR principles. While [5,14,19] mentioned clustering analysis, there is no way to judge based on these publications which clustering approach is most effective. To identify influential publications, ref. [5] measured node centrality; ref. [14] measured popularity. To identify similar papers, ref. [5] used nearest neighbors on RDF2vec embeddings, whereas [14], besides using SciBERT embeddings, also defined novel metrics such as topic and citation similarity. Again, there is no way to assess which approach would be more beneficial to a user. As mentioned previously, a unique and useful feature of [19] is ACTA, which allows the creation of argumentative graphs and identifying PICO elements. Surprisingly, the authors of [19] did not include any results in their publication showing the utility of such argumentative graphs, for example, by surveying a group of clinicians and reporting their user experience. When we used ACTA to check its PICO identification capability, we found qualitatively that it performs well at identifying the PICO elements, except for the *P* (patients/population) element. We are unsure whether this is because the publicly available search engine does not utilize the latest version of their code, or because ACTA is not trained to detect the *P* element.

During the peer review process, we were made aware of a paper by Giarelis et al. [36] that is more rigorous in its performance evaluation than the papers included in this review. It was not found in our search, as it is not indexed on PubMed, and our Google and Google Scholar searches were limited to the first 50 results. The paper uses KGs to address the problem of discovering future research collaborations. The authors treat it as a binary classification problem, which allows them to report standard metrics such as accuracy, precision, and recall. A sample is labeled as positive or negative depending on the presence or absence of a *co_authors* edge between two *Author* nodes. For all their performance evaluation experiments, they used subsets of the CORD-19 dataset, which is the obvious choice for a benchmark dataset. Using such a standard dataset to address a well-defined question and reporting widely used performance metrics would make quantitative comparisons between papers possible.

### 4.2. Knowledge Graphs for Drug Repurposing

Five papers were included in this application group: Stebbing et al. [21], Wang et al. [23], Domingo-Fernandez et al. [24], Hsieh et al. [25], and Zhou et al. [26]; the last of these is a review article. Stebbing et al. [21] is a comment article that focuses on a proprietary AI algorithm. The article makes the output of the AI algorithm explainable: using clear logic that dispels any notions of a black box, the authors justify why baricitinib should be an effective treatment for COVID-19, a claim that has been verified in a clinical trial. However, as the algorithm is proprietary, there is no explanation about how it works. Wang et al. [23] is the only paper in this review that talks about drug repurposing report generation. The content of such an AI-generated drug report, mentioned in Section 3.2, is very useful to understand why a drug repurposing candidate was chosen. The paper mentions that the reports were reviewed by clinicians and medical students, but a more quantitative appraisal will perhaps be available at a later stage. The paper is also unique in using figure images from publications to enrich their KG.

The most notable feature of the KG in [24] is that it is human-curated. While the present-day superiority of human curation is hard to deny, it is noteworthy that their KG only contains information from 160 original research articles. It is unclear whether human curation would be sustainable if this number were one or two orders of magnitude higher. Their method was able to identify over 300 candidate drugs being investigated to treat COVID-19. By contrast, ref. [25] starts by considering all drugs (*n* = 3635) being investigated for COVID-19 treatment. Another key difference between [24] and [25] is that [24] uses a web application to allow users to query, browse, and navigate their KG, whereas [25] presented a list of top repurposable drugs. As a result, ref. [25] seems more lay-user-friendly, but that does not mean that its top-ranked drugs will actually be effective treatments for COVID-19.

### 4.3. Multi-Purpose Knowledge Graphs

Three papers were included in this application group: Chen et al. [30], Reese et al. [33], and Ostaszewski et al. [34]. Chen et al. [30] discussed four experiments in their paper: identifying experts on coronavirus topics for building collaborations, named entity recognition with BioBERT, co-occurrence frequency-based KG, and cosine similarity-based KG. Their results for BioBERT showed a named entity recognition that is far worse than human (best F1-scores around 0.75) but had the advantage of parsing far more documents than humanly possible. It is unclear if this trade-off is worthwhile. While their two KG experiments can be used for drug repurposing and produces pleasing figures, an example of which is shown in Figure 7, that approach is far less sophisticated than some of the papers mentioned under the drug repurposing application group [23,24,25].

Reese et al. [33] downloaded data from multiple siloed and incompatible data sources before converting and combining them using the KGX interchange format. It is unclear why KGX is not a more widely used format: it was not mentioned in any of the other papers included in this review, despite offering the advantage of combining features of RDF and property graphs. To create KG embeddings, they used Embiggen instead of the more widely used RDF2vec. As a manuscript describing Embiggen is in preparation, it is not possible for us to evaluate its potential advantages over RDF2vec. The authors used these embeddings to address the machine learning problem of link prediction: drug to disease, drug to gene, and drug to protein. While they qualitatively showed the utility of their approach using *t*-SNE plots, no quantitative results using metrics such as accuracy or F1-score were presented.

Ostaszewski et al. [34] summarized the massive effort undertaken by the COVID-19 Disease Map community to create an open-access collection of curated computational diagrams and molecular mechanisms models implicated in COVID-19. The Disease Map is a collection of 41 such diagrams containing 1836 interactions between 5499 elements, supported by 617 articles. While the authors do create KGs, they mention that KGs have a broad coverage at the cost of depth of mechanistic interpretation. Hence, the goal of these KGs is to allow biocurators to enrich the previously mentioned pathway diagrams, making human–machine collaboration a reality. The end users of such diagrams are bioinformaticians and computational biologists, rather than clinicians.

## 5. Conclusions

In this work, we have provided an exploratory review on KGs in the context of COVID-19. By providing links between disparate datasets that are stuck in silos, KGs enable the user to effectively search the overwhelming volume of COVID-19 research and gain actionable insight which would either be extremely tedious or impossible to achieve in the absence of such emerging uses of AI. We believe that in these early days of KGs, it is difficult to fully assess the potential of each work, as the results they have presented are in most cases preliminary. We see the main contribution of our paper as being a detailed summary of various approaches rather than their ultimate performance, with the aim of raising awareness of the technology behind KGs.

## Figures and Tables

**Table 1:** Summary of papers resulting from our literature curation.

Authors	Title	Application
Kejriwal	Knowledge Graphs and COVID-19: Opportunities, Challenges, and Implementation	KG overview
Steenwinckel et al.	Facilitating the analysis of COVID-19 literature through a knowledge graph	Literature search
Wise et al.	COVID-19 knowledge graph: accelerating information retrieval and discovery for scientific literature	Literature search
Cernile et al.	Network graph representation of COVID-19 scientific publications to aid knowledge discovery	Literature search
Michel et al.	COVID-on-the-Web: Knowledge graph and services to advance COVID-19 research	Literature search
Stebbing et al.	COVID-19: combining antiviral and anti-inflammatory treatments	Drug repurposing
Wang et al.	COVID-19 literature knowledge graph construction and drug repurposing report generation	Drug repurposing
Domingo-Fernandez et al.	COVID-19 Knowledge Graph: a computable, multi-modal, cause-and-effect knowledge model of COVID-19 pathophysiology	Drug repurposing
Hsieh et al.	Drug Repurposing for COVID-19 using Graph Neural Network with Genetic, Mechanistic, and Epidemiological Validation	Drug repurposing
Zhou et al.	Artificial intelligence in COVID-19 drug repurposing	Drug repurposing
Chen et al.	Coronavirus knowledge graph: A case study	Multi-purpose
Reese et al.	KG-COVID-19: a framework to produce customized knowledge graphs for COVID-19 response	Multi-purpose
Ostaszewski et al.	COVID-19 Disease Map, a computational knowledge repository of SARS-CoV-2 virus–host interaction mechanisms	Multi-purpose
Bettencourt-Silva et al.	Exploring the Social Drivers of Health During a Pandemic: Leveraging Knowledge Graphs and Population Trends in COVID-19	Risk factor discovery

## Supplementary Materials

The following are available online at https://www.mdpi.com/article/10.3390/jpm11040300/s1, Figure S1: Framework used by Wang et al [23], Figure S2: Expanding KG through Subfigure Segmentation and Cross-modal Entity Grounding.
