# Supplementary material for: Knowledge Graphs for COVID-19: An Exploratory Review of the Current Landscape
Source: J Pers Med. 2021 Apr 14;11(4):300. doi: 10.3390/jpm11040300 (PMC8070774; doi:10.3390/jpm11040300)
Supplement: Supplementary file 1 [file jpm-11-00300-s001.zip › Supplementary.docx]

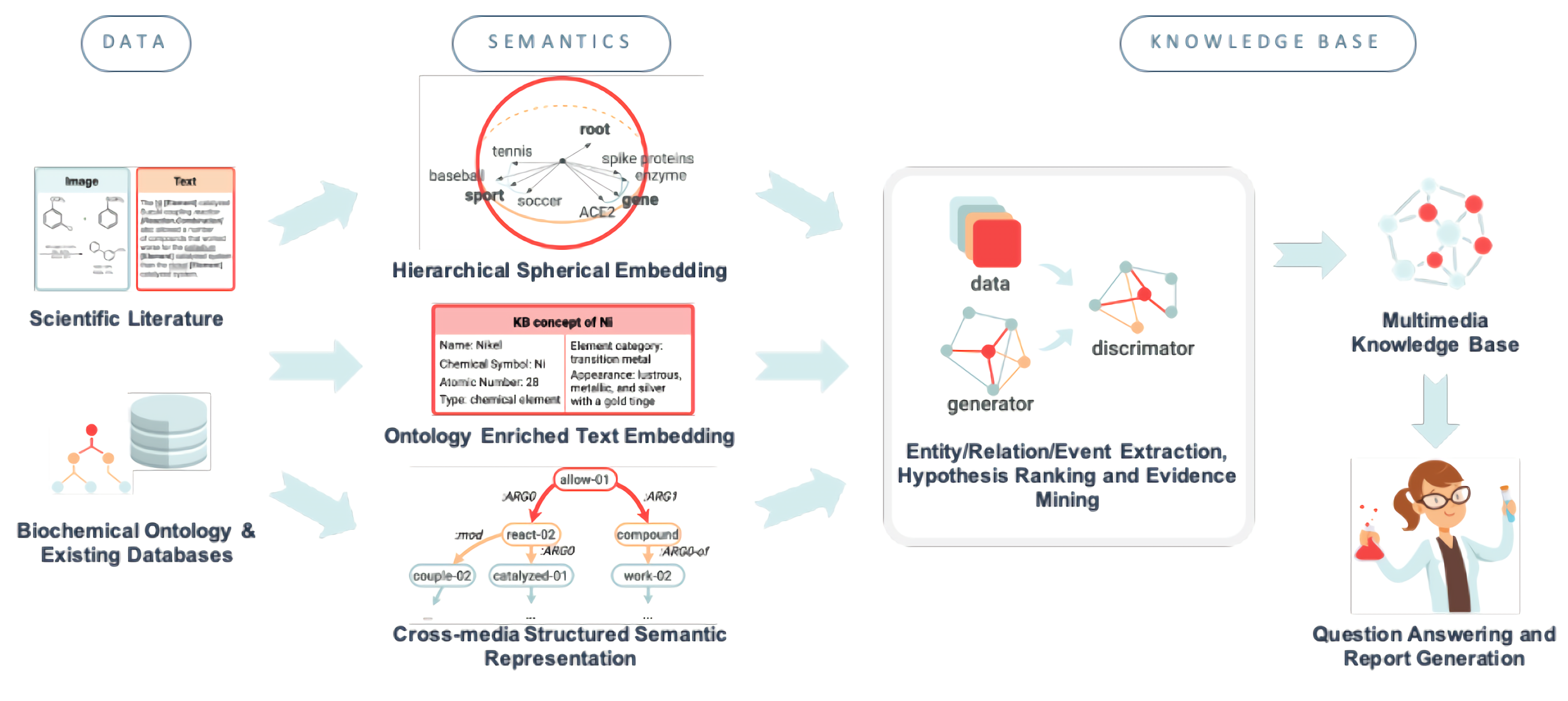


**Figure S1.** Framework used by Wang et al [23]. Reproduced with permission.


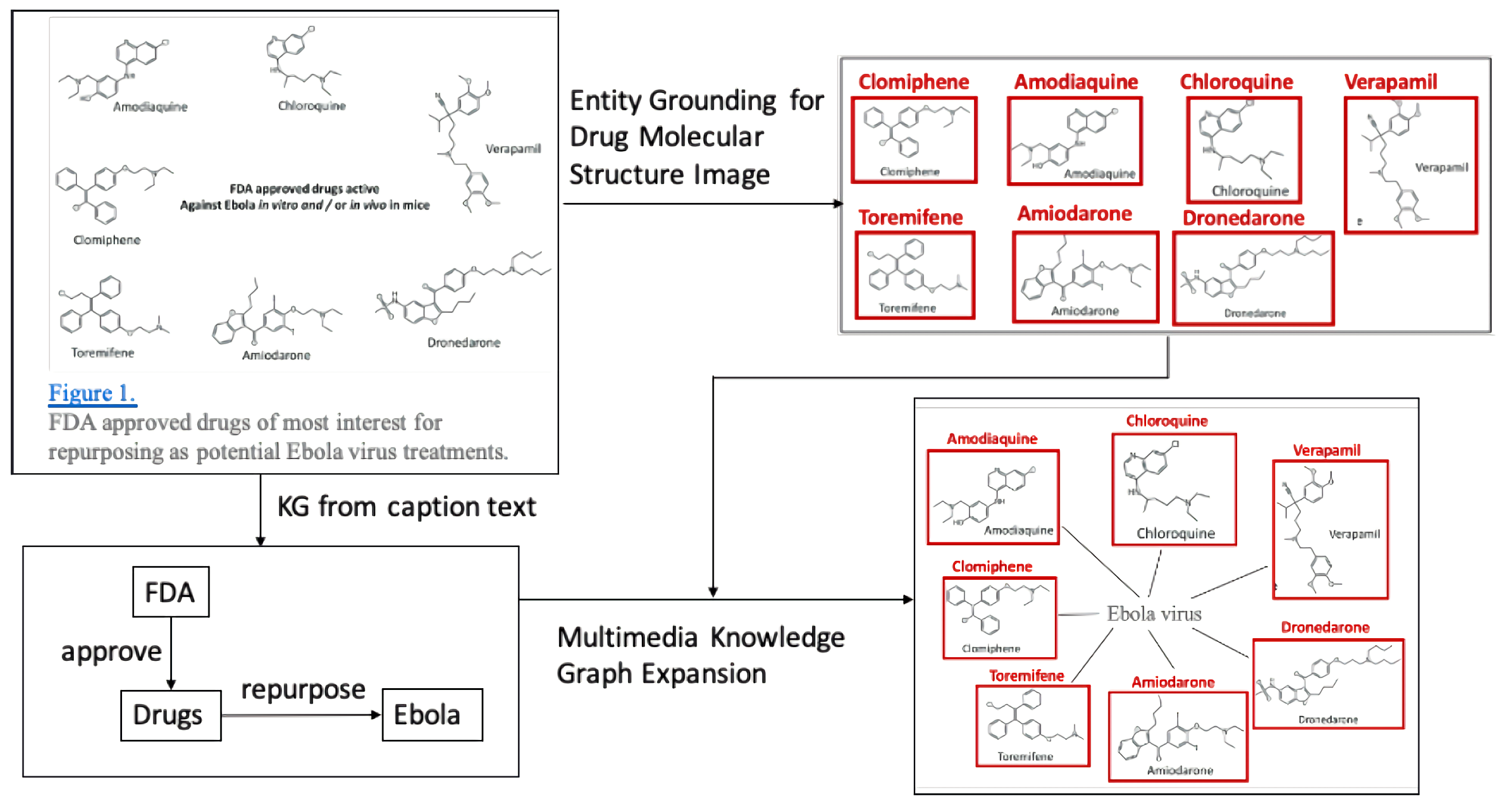


**Figure S2.** Expanding KG through Subfigure Segmentation and Cross-modal Entity Grounding. Reproduced with permission from Wang et al [23].
